# FuncPEP: A Database of Functional Peptides Encoded by Non-Coding RNAs

**DOI:** 10.3390/ncrna6040041

**Published:** 2020-09-23

**Authors:** Mihnea P. Dragomir, Ganiraju C. Manyam, Leonie Florence Ott, Léa Berland, Erik Knutsen, Cristina Ivan, Leonard Lipovich, Bradley M. Broom, George A. Calin

**Affiliations:** 1 Department of Translational Molecular Pathology, The University of Texas MD Anderson Cancer Center, Houston, TX 77030, USA; leonieflorence.ott@gmail.com (L.F.O.); leaberland370@gmail.com (L.B.); erik.knutsen@uit.no (E.K.); civan@mdanderson.org (C.I.); 2 Department of Surgery, Fundeni Clinical Hospital, Carol Davila University of Medicine and Pharmacy, 022328 Bucharest, Romania; 3 Department of Bioinformatics and Computational Biology, The University of Texas MD Anderson Cancer Center, Houston, TX 77030, USA; GCManyam@mdanderson.org (G.C.M.); BMBroom@mdanderson.org (B.M.B.); 4 Institute of Tumor Biology, University Medical Center Hamburg-Eppendorf, 20246 Hamburg, Germany; 5 Department of Medical Biology, Faculty of Health Sciences, UiT—The Arctic University of Norway, N-9037 Tromsø, Norway; 6 Center for RNA Interference and Non-Coding RNAs, The University of Texas MD Anderson Cancer Centre, Houston, TX 77054, USA; 7 Center for Molecular Medicine and Genetics, Wayne State University, Detroit, MI 48201, USA; llipovich@med.wayne.edu

**Keywords:** non-coding RNAs, long non-coding RNAs, ncRNA-encoded peptides, small open reading frames, ncRNA translation, small peptides, micropeptides

## Abstract

Non-coding RNAs (ncRNAs) are essential players in many cellular processes, from normal development to oncogenic transformation. Initially, ncRNAs were defined as transcripts that lacked an open reading frame (ORF). However, multiple lines of evidence suggest that certain ncRNAs encode small peptides of less than 100 amino acids. The sequences encoding these peptides are known as small open reading frames (smORFs), many initiating with the traditional AUG start codon but terminating with atypical stop codons, suggesting a different biogenesis. The ncRNA-encoded peptides (ncPEPs) are gradually becoming appreciated as a new class of functional molecules that contribute to diverse cellular processes, and are deregulated in different diseases contributing to pathogenesis. As multiple publications have identified unique ncPEPs, we appreciated the need for assembling a new web resource that could gather information about these functional ncPEPs. We developed FuncPEP, a new database of functional ncRNA encoded peptides, containing all experimentally validated and functionally characterized ncPEPs. Currently, FuncPEP includes a comprehensive annotation of 112 functional ncPEPs and specific details regarding the ncRNA transcripts that encode these peptides. We believe that FuncPEP will serve as a platform for further deciphering the biologic significance and medical use of ncPEPs. The link for FuncPEP database can be found at the end of the Introduction Section.

## 1. Introduction

Large-scale transcriptomics efforts, such as the FANTOM Consortium [1], have revealed that most of the mammalian genome is pervasively transcribed. Most of these transcripts are classified as non-coding RNAs (ncRNAs) [2], and over two-thirds of human genes are, essentially, transcribed solely into ncRNAs that do not encode known proteins [3,4]. NcRNAs are defined as transcripts that lack an open reading frame (ORF), in particular those lacking ORFs of >100 amino acids (aa) in size and devoid of ORF evolutionary conservation [5]. More recently, some ncRNA transcripts have been documented by experimental methods, such as mass spectrometry (MS), to encode previously unknown short peptides [6,7].

The common definition of an ncRNA coding for peptides is that of a transcript, initially annotated as non-coding, that subsequently was identified as containing a small open reading frame (smORF) encoding a peptide of less than 100 aa [8]. This definition is consistent with the fact that >95% of conventional known proteins from public databases are longer than this threshold. NcRNA-encoded peptides (ncPEPs) can be predicted by multiple computational methods [5,9] but ultimately require biological validation at the level of their biogenesis—which can be confirmed indirectly by ribosome profiling [10] or directly by MS [6]. Biologically, it is still uncertain if the function, biogenesis, or structural properties of ncPEPs are different from “classic” coding region-derived peptides. In comparison to protein-coding genes, and to short conserved ORFs of known functional peptides encoded by a subset of protein-coding genes, smORFs in ncRNAs lack evolutionary conservation and, without laboratory-based validation, also lack bioinformatics evidence of function [11]. Furthermore, many ncRNAs, and hence their respective ncPEPs, are expressed in a tissue- and species-specific manner [3]; this suggests distinct, perhaps essential, organism- or evolution-specific functions. Indeed, many of the ncPEPs have recently emerged as functional and possess newly characterized fundamental roles in cellular processes and the maintenance of cellular homeostasis [12,13]. The biogenesis of ncPEPs seems to differ from the other peptides as they are often translated from unique small coding sequences, smORFs, while peptides from coding regions are translated from well-known mRNAs that are phylogenetically conserved or are formed through the cleavage of larger peptides/proteins [14]. In particular, human smORFs may lack conservation beyond primates, whereas conventional human proteins typically exhibit at least pan-vertebrate, and sometimes pan-metazoan, conservation [15,16]. The sequence of an ncRNA smORF usually begins with a traditional AUG start codon and frequently does not terminate with typical stop codons. This start AUG codon (ATG codon in cDNA), by using ORF-finding software (such as the NCBI ORF Finder [17] or other similar tools [18]), permits the identification of hypothetical smORFs from various genomic locations, including regions annotated as non-coding. Thousands of potential small peptides have already been predicted in different organisms, but most of these arise from en masse computational identification attempts that automatically find all possible ORFs. Many of these potential peptides are translated from transcripts that had been annotated as ncRNAs, and only for a very few has their potential expression and function been studied. With the development of new identification techniques, we expect that the number of functionally validated ncPEPs will rapidly increase.

Therefore, we posit that a curated database of functionally confirmed peptides arising from ncRNA transcripts is highly necessary. Currently, our database, named **fu**nctional **nc**RNA encoded **pep**tides (FuncPEP), contains 112 peptides encoded by ncRNAs, all of which have been validated indirectly by ribosome profiling of the corresponding “host” ncRNAs, loss-of-function techniques, and/or directly by MS, Western blotting or immunostaining, and are biologically functional, being linked to a physiological or a pathological phenomenon. We decided to include indirectly confirmed ncPEPs, which we hope will be confirmed directly in the future. We are confident that discoveries in upcoming years will allow us to widely expand this database.

NcRNAs are commonly classified as long ncRNAs (lncRNAs) and short non-coding RNAs (sncRNAs). LncRNAs, in contrast to sncRNAs, are defined by a length of >200 nt. Both classes of ncRNAs consist of several different ncRNA species. While long-intergenic ncRNAs (lincRNAs) or transcribed ultraconserved regions (T-UCRs) are part of the lncRNA class, microRNAs (miRNAs), transfer RNAs (tRNAs), and PIWI-interacting RNAs (piRNAs) are examples of sncRNAs. NcRNA species of various lengths, such as circular RNAs (circRNAs) or small nucleolar RNAs (snoRNAs), can be assigned to both classes of ncRNAs in dependence of their specific size [19,20]. It is widely appreciated that most ncRNA classes, such as circRNAs, miRNAs, and lncRNAs, have distinct, important, and, in many cases, essential functions [21,22,23]. In cases where these ncRNAs contain experimentally validated smORFs, the ncRNAs functions may be different and independent from, or in exceptional cases even opposite to, the functions of the encoded peptide [24]. Alternatively, the smORF translation may be a non-functional byproduct of the ncRNAs’s transcript, and all essential functions might still be carried out solely at the RNA level.

During our systematic literature review, we noticed several intriguing cases where a transcript initially annotated as an ncRNA that encodes a peptide was reclassified as a protein-coding transcript. This exposes several fundamental and paradoxical conundrums of post-genomic biology: Are “ncRNAs” really biologically, and empirically, non-coding? How many ncRNAs are translated into peptides? Are some ncRNAs incorrectly annotated in transcriptome databases, being actually protein-coding transcripts as only the coded peptide is functional, or do such ncRNAs harbor both non-coding transcript and small peptide functions? Are some functional ncRNAs only occasionally, unexpectedly and erroneously translated by cellular ribosomes?

More than a decade has passed since these conundrums were raised for the first time, and no definitive answers exist [5]. Clearly, a subset of “lncRNA translation” events is an annotation artifact, and is due to the erroneous mis-annotation of certain mRNAs as “lncRNAs” [7]. Two distinguishing properties characterized this subset: Robust translation at levels comparable to those of other highly expressed known protein-coding genes, and obvious ORF homologies to known proteins in multiple species. Furthermore, considerable annotation ambiguities exist. Certain genes, such as steroid receptor RNA activator (SRA), are inherently bi-functional, with well-validated functions as both ncRNA transcript and as a peptide-codifying gene [25]. This shows that the binary division of transcripts into coding and non-coding could be in some instances a “false dichotomy”. Recently, lncRNA proteogenomics was developed, by using direct MS methods, rather than indirect computational or lab-based methods. All human lncRNA genes from the ENCODE Consortium’s Gencode human gene catalog were tested for evidence of translation in one normal cell type and one cancer cell line. It was determined that most lncRNAs are very rarely translated, as only approximately 1% of lncRNAs have smORFs whose translation is detectable by MS. However, many of these translational events were singletons (and therefore possible false positives or rare cellular mistakes), and lncRNAs were vastly translationally depleted, compared to known mRNAs, even after normalization for expression-level differences. Intriguingly, a few peptides were “non-singleton hits” (detected as multiple independent events in the mass spectra, meaning that they were real translation products and not one-off artifacts), suggesting that MS is a valuable approach for direct discovery of translated lncRNA ORFs [7]—a direct approach that remains persistently underutilized in an era of continuing overreliance on indirect ribosome-profiling-based detection of putative lncRNA translation.

We constructed a new database in order to centralize and monitor this valuable information, to systematically represent the ncRNAs and the corresponding ncPEPs’ main characteristics, and to facilitate the use of this information for precision medicine and post-genomic era therapeutics. The database can be accessed at https://bioinformatics.mdanderson.org/Supplements/FuncPEP/.

## 2. Data Collection and Computational Methods

### 2.1. Data Collection and Database Construction

The information stored in our database was obtained through a systematic literature search and review from the NCBI PubMed and Google Scholar databases for the period 28 May 1996, when the first paper describing an ncPEP was published [26] to present (July, 2020). The literature review was conducted by two independent researchers (L.F.O. and L.B.) and disagreements were resolved by discussion with two other investigators (M.P.D. and C.I.). We selected peptides accurately identified to be encoded by ncRNAs by combining the following search terms: At least one of the following terms regarding non-coding transcripts (“antisense RNA,” OR “lincRNA”, OR “lncRNA”, OR “miRNA”, OR “circRNA”, OR “rRNA”, OR “tRNA”, OR “ncPEP”) AND at least one of the following terms regarding translation (“smORF,” OR “Ribo-seq”, OR “ribosome profiling”, OR “mass spectrometry”, OR “translation”). Furthermore, additional manual search of the published ncRNA literature was regularly performed by a fifth author (G.A.C.).

Three additional filters were employed, and we currently require full compliance with all three conditions:

(i) We included only ncPEPs that were directly (MS, Western blotting, immunohistochemistry, immunofluorescence) or indirectly (ribosome profiling, loss/gain of function studies) experimentally confirmed;
(ii) All included ncPEPs were not merely experimentally confirmed but also functionally characterized (linked to a physiological or a pathological process); and
(iii) For concordance with the definitions discussed above, we considered ncPEPs of only ≤ 100 aa in length.

NcPEPs confirmed by indirect methods are marked by an asterisk “*” in the database and will be screened for further confirmation by direct methods. They currently comprise a major part of the database because ribosome profiling studies (also known as “Ribo-seq”, i.e., selective sequencing of only the ribosome-bound RNA fraction instead of all cellular RNAs) remain more prevalent than MS. Of note, solely computationally predicted ncPEPs are not included in the database but will be added if experimentally confirmed.

The FuncPEP database includes the peptide’s name assigned by the authors, its symbol, the name assigned by NCBI, as well as the synonyms that we were able to identify. In accordance with data provided by authors, we also extracted the peptides’ lengths, molecular weight, sequence, functions, and data about the related ncRNA. In addition, we included data and links from two NCBI portals, Protein and Gene, and links to the Pfam 33.1 database providing predicted protein domains of the ncPEPs, giving additional potential insights into the function of ncPEPs.

As vast sequence data is available in the Pfam protein database, deep learning was utilized to build the prediction model using Python 3.4. The Pfam seed sequence data with over 1.25 million records was divided into training (80%) and testing sets (20%). Keras 2.3.0 with TensorFlow 2.0. was used on this sequential data with a variant of convolution neural network called the residual convolution network to identify the functional classes of proteins [27]. Only families with at least 10 peptide sequences in the database were used in building the model to predict domains of peptides from ncRNA. The final convolution network was generated with a batch size of 256 running for 20 epochs. This residual convolution network model performed well on test data with an accuracy of over 95%. The Pfam domains for non-coding peptides of lengths greater than 20 were predicted using this neural network model. The corresponding domain information is included in the FuncPEP database.

We assigned individual identifiers to the retrieved peptides, as symbols and names utilized across the research community might become ambiguous over time. The website for FuncPEP database was developed using R open source programming language and formatted by Markdown.

### 2.2. Sequence and Molecular Weight Prediction

We collected all the given information from each paper. However, sometimes information, such as the aa sequence or molecular weight, was not given. In order to provide a complete database, we used the NCBI ORF Finder (https://www.ncbi.nlm.nih.gov/orffinder/) to predict amino acid sequences when only the DNA sequence of the respective ORF was provided. Additionally, we used the Peptide Molecular Weight Calculator by Selleckchem (https://www.selleckchem.com/peptide-calculator.html) to calculate the molecular weights of peptides without provided weight or when only the approximate weights derived from Western blots were given.

### 2.3. Comparison of ncPEPs with Known Peptides Encoded by Messenger RNAs of Coding Genes

We compared the composition of human ncPEPs with that of human peptides from coding regions. For this purpose, we downloaded from https://db.systemsbiology.net/sbeams/cgi/PeptideAtlas/buildInfo the sequences of 2,476,065 distinct human tryptic-digest peptides detected by mass spectrometry analysis of several human organs and datasets (all ≤ 100 aa), build Human 2020–01. Several criteria for classifying human peptides from a biochemical perspective were retrieved from [26,27]. The composition of amino acids, respectively, classes of amino acids in peptides sequences, was obtained with Perl and R (version 3.5.1) codes.

The significance of the difference between the proportions of different amino acids in the human peptides encoded by messenger RNAs compared to human ncPEPs was assessed in R (version 3.5.1) with the nonparametric Mann–Whitney–Wilcoxon test with a default correction method Benjamini & Hochberg for multiple testing and with a Chi-Square test. A box-and-whisker plot (Box plot represents first (lower bound) and third (upper bound) quartiles, whiskers represent 1.5 times the interquartile range) was used to visualize the data.

## 3. Implementation and Results

### 3.1. Systematic Review

**Figure 1:**
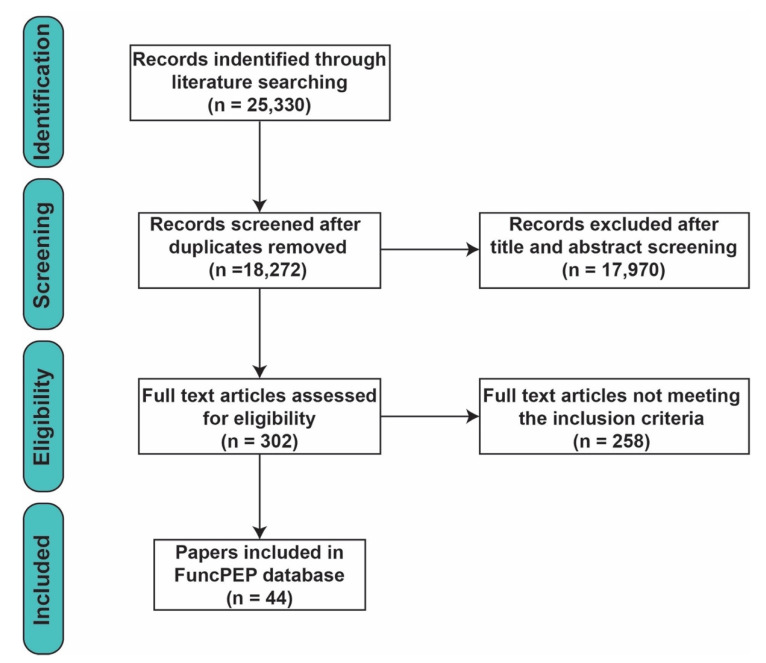
Flowchart describing the process of scientific paper identification, screening, eligibility testing, and inclusion.

A total of 25,330 articles were identified on PubMed and Google Scholar databases. After removing the duplicates, 18,272 articles were screened by title and abstract, and 302 articles were considered for full-text assessment. Of these, 44 studies, containing 112 validated functional peptides, were included in FuncPEP′s database according to our inclusion criteria (Figure 1).

### 3.2. Database Interface

FuncPEP provides a user-friendly interface through a website allowing access to an ncPEP database. The database website can be accessed at: https://bioinformatics.mdanderson.org/Supplements/FuncPEP/.

**Figure 2:**
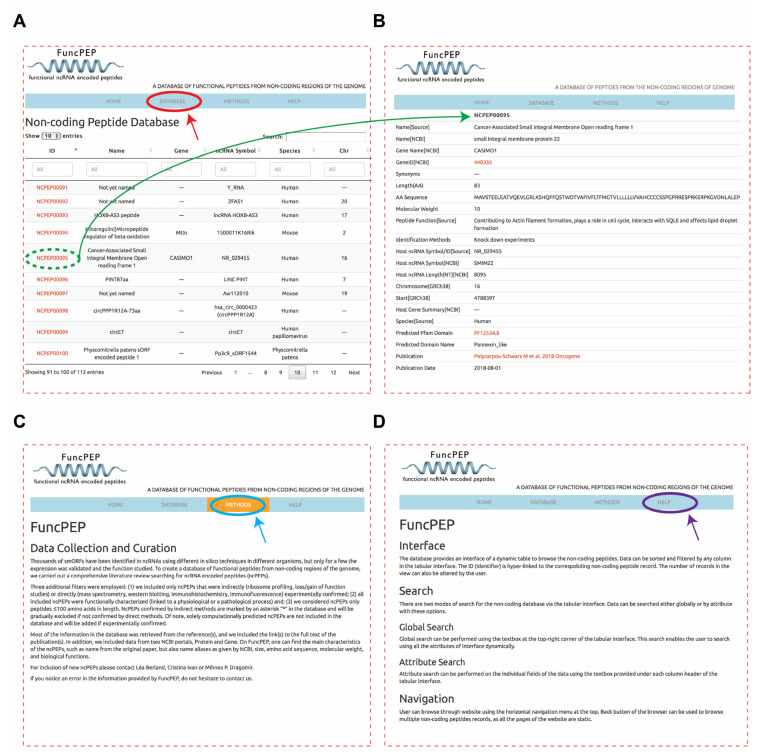
Database interface (**A**) The database section of FuncPEP providing an overview of information provided for each ncPEP. (**B**) Complete information for a selected ncPEP, accessible through a dynamic table browser. (**C**) The methods section of FuncPEP, describing the methodology used to curate and collect the ncPEPs data. (**D**) The help section of FuncPEP containing information on how to navigate the site.

**Figure 3:**
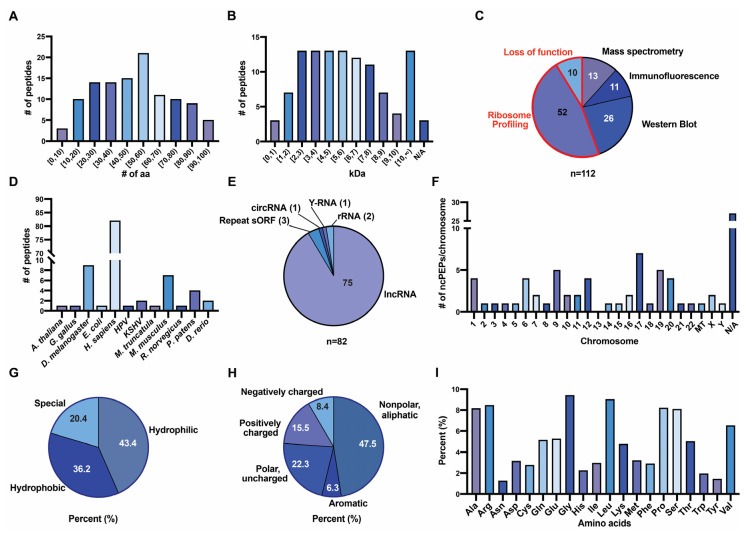
Characteristics of ncPEPs from the FuncPEP database. (**A**) NcPEP´s length in amino acids; (**B**) NcPEP´s molecular weight (kDa); (**C**) Methods used to detect ncPEPs (red border and font—indirect identification methods, black border and font—direct identification methods); (**D**) Species in which ncPEPs were discovered; (**E**) Classification of human ncPEP´s host ncRNA; (**F**) Chromosomal distribution of human ncPEPs; (**G**) Classification of human ncPEP´s amino acids into hydrophobic, hydrophilic, and special; (**H**) Classification of human ncPEP´s amino acids according to the chemistry of the R groups; (**I**) Amino acids composition of human ncPEPs. #: ncPEPs.

The database website is divided into four sections: (1) The Home section, where users can find background information on the database as well as on the topic of ncPEP; (2) the Database section (red circle and arrow), where all information on ncPEPs is accessible through a dynamic table browser and providing an overview of the ncPEPs (Figure 2A) and complete information for each ncPEP can be accessed by selecting the respective ncPEPs ID (green dashed circle and arrow) (Figure 2B); (3) the Methods section (blue circle and arrow), describing the methodology used to curate and collect the ncPEPs data (Figure 2C); and (4) the Help section (purple circle and arrow), containing information on how to navigate the site (Figure 2D).

### 3.3. Characteristics of ncPEPs from the FuncPEP Database

FuncPEP was created as a useful resource for accumulating ncPEP data from published scientific literature. One of FuncPEP’s aims is to provide comprehensive information about functionally confirmed ncPEPs. To establish a clear overview of the topic, FuncPEP also contains information about corresponding ncRNAs: their length, the genomic position, symbols from the source and NCBI, and the predicted Pfam domain.

In accordance, ncPEPs included in FuncPEP are all less than 100 aa. The average size of the 112 ncPEPs included in FuncPEP is about 49 aa (Figure 3A). FuncPEP also contains the molecular weight of the retrieved ncPEPs; for human ncPEPs, this ranges between 0.65 and 15 kDa, with an average of 5.7 kDa (Figure 3B).

Most of the peptides in our database were not detected by a single but by several different methods. Often, bioinformatically predicted smORFs located in ncRNA were experimentally proven to be translated. Since bioinformatics predictions were regarded as insufficient proof for the existence of an ncPEP, only experimentally confirmed peptides were included. Experimental methods range from indirect ones (red border), such as loss-of-function assays and ribosome profiling, to direct ones (black border), like immunofluorescence staining, Western blotting, or MS, the latter providing the highest sensitivity. Only taking the most reliable detection method into consideration, 13 (11.6%) of the peptides in our database were detected by MS, 26 (23.2%) by Western blot, 11 (9.8%) by immunostaining methods, 52 (46.4%) by ribosome profiling, and 10 (8.9%) by loss-of-function studies (Figure 3C).

Next, we analyzed in which species the ncPEPs were discovered. Importantly, most ncPEPs have been discovered and studied in *Homo sapiens* (73.2%, 82/112), followed by *Drosophila melanogaster* (8%, 9/112) and *Mus musculus* (6.3%, 7/112) (Figure 3D). Some ncPEPs can be found across different species, such as Myoregulin identified in *Homo sapiens* and *Mus musculus*, or Humanin, detected in *Homo sapiens* and *Rattus norvegicus*. This implies a certain degree of conservation amongst some ncPEPs, and strengthens the case for their potential functionality, even though for lncRNAs at large and presumably for their smORFs—lack of conservation does not imply lack of function [28]. Yet, because the majority of ncPEPs was discovered in humans, we decided to further focus on characterizing human ncPEPs.

We analyzed the biotypes of ncRNAs that harbor human ncPEP smORFs. The majority of human peptides are encoded by lncRNAs (91.5%, 75/82), three by smORFs in genomic GGGGC repeats (3.7%), two by mitochondrial rRNAs (2.4%), and one each by a Y-RNA (1.2%) and a circRNA (1.2%) (Figure 3E). This data underlines the fact that sncRNA only exceptionally are encoding ncPEPs and usually the longer immature forms of sncRNAs are the ones long enough to contain ncPEP smORFs. The best-known examples of sncRNA-encoded peptides were discovered in plants and are encoded by two pri-miRNAs: pri-miR-171b (*Medicago truncatula*) and the pri-miR-165a (*Arabidopsis thaliana*), which are known to produce specific peptides miPEP171b and miPEP165a [29]. While in theory all ncRNA are a potential source of ncPEPs and our database contains ncPEPs from multiple ncRNA species, the majority of ncPEPs are derived from lncRNAs. Of note, having a length of up to several hundreds of nucleotides, most circRNAs and pri-miRNAs are also lncRNAs [30,31].

Subsequent analysis of the chromosomes that harbor the respective human ncRNAs revealed that, except for chromosome 13, ncPEPs can be found on all chromosomes, including the X and Y chromosomes (Figure 3F). The potential lack of ncPEPs on chromosome 13 (although we cannot exclude the identification of ncPEP from chromosome 13 ncRNAs), is likely due to the gene-poor nature of this chromosome (this is also the reason why trisomy 13, 18, or 21 are the only live birth survivable trisomies in humans): The fewer genes there are in general, the fewer ncRNAs and hence ncPEPs. Indeed, we also observed only one ncPEP on chromosome 18 and one on chromosome 21.

Furthermore, ncPEPs’ structural characteristics presented in the FuncPEP database include all data considered relevant and useful for peptide studies. Hence, we evaluated ncPEPs in regard to their aa chemistry and composition. All the following analyses are taking into consideration only human ncPEPs with a confirmed aa sequence (*n* = 31). Analyzing the composition [32] of curated ncPEPs, we found that they are similarly composed of hydrophilic (43.4%) and hydrophobic amino acids (36.2%) and the remaining 20.4% are special ones (glycine, cysteine, proline) (Figure 3G). Moreover, evaluating the aa´s charge [33] of ncPEPs, 47.5% of the aa carry non-polar aliphatic R-groups, 6.3% aromatic, 22.3% polar uncharged, 15.5% positively charged, and 8.4% negatively charged R-groups (Figure 3H). Looking more in detail at ncPEPs’ aa composition, we observed that glycine, leucine, arginine, proline, and alanine are the five most frequent aa from the structure of human ncPEPs accounting for 43% of all aa, whereas tryptophan, tyrosine, and asparagine are the rarest ones with less than 5% in total (Figure 3I).

### 3.4. Comparison of Human Functional ncPEPs with Human Peptides from Coding Regions

**Figure 4:**
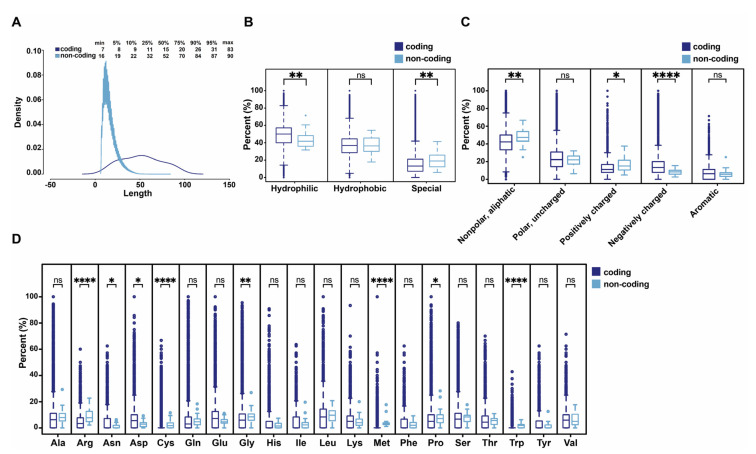
Up-to-date comparison of human functional ncPEPs with human peptides from coding regions. (**A**) Comparison of the size range between human ncPEPs and peptides from coding regions. (**B**) Comparison between human ncPEPs and peptides from coding regions according to the properties of the amino acids (hydrophobic, hydrophilic, and special). (**C**) Comparison between human ncPEPs and peptides from coding regions according to the chemistry of the R groups (aromatic R groups; nonpolar aliphatic R groups; negatively charged R groups; positively charged R groups; and polar uncharged groups). (**D**) Comparison between human ncPEPs and peptides from coding regions according to the amino acid distribution. Due to a not normally distributed dataset, data is presented using box plots with whiskers representing 1.5 times the interquartile range. (Mann–Whitney–Wilcoxon test with Benjamini & Hochberg correction for multiple testing; ns—not significant, * *p* < 0.05; ** *p* < 0.01; **** *p* < 0.0001).

Subsequently, we compared the physico-chemical properties of human ncPEPs with an aa sequence provided (*n* = 31) with human tryptic-digest peptides detected by MS and mapping to known protein-coding regions (*n* = 2,476,065) derived from the PeptideAtlas (https://db.systemsbiology.net/sbeams/cgi/PeptideAtlas/buildInfo, [34]). Despite the significantly different sizes of the datasets, we first compared the length of the human ncPEPs with the sequence provided and the human coding peptides. NcPEPs range from 16 to 90 aa in length, with a median length of 52 aa. Human coding peptides range from 7 to 83 aa in length with a median length of 15 aa (Figure 4A). Furthermore, we analyzed the chemical properties of the aa and noticed a similar content of hydrophobic aa across both peptide groups (*p* = n.s.). However, they differed significantly in regard to their hydrophilic and special aa content. The ncPEPs contain less hydrophilic (*p* = 0.0064) but more special aa (*p* = 0.0037) (Figure 4B). Moreover, we also noticed several differences between the two groups of peptides regarding the chemistry of their R-groups. NcPEPs have a significantly more nonpolar aliphatic group (*p* = 0.0072) and positively charged groups (*p* = 0.0115) compared to peptides from coding regions. On the other hand, ncPEPs contain significantly less negatively charged R-groups compared to human peptides from coding regions (*p* = 3.36 × 10^−5^). Only the content of aromatic R-groups (*p* = n.s.) and of polar uncharged R-groups (*p* = n.s.) is similar between the two groups (Figure 4C).

Additionally, we compared the distribution of aa on the peptides within the two sample sets. In human peptides encoded by coding RNAs, the three most abundant aa are leucine, glutamic acid, and serine while methionine, cysteine, and tryptophan are all rather rare aa [32]. Human ncPEP´s aa composition displays some parallels to, but is mainly distinct from, the composition of the peptides from coding regions. NcPEPs contain more arginine (*p* = 3.59 × 10^−7^, cysteine (*p* = 7.82 × 10^−6^), glycine (*p* = 0.0031), methionine (*p* = 8.81 × 10^−14^), proline (*p* = 0.0272), and tryptophan (*p* = 1.38 × 10^−13^) compared to human peptides from coding regions. In contrast to that, the prevalence of asparagine (*p* = 0.0296) and aspartic acid (*p* = 0.0196) is significantly lower in ncPEPs. The distribution of the other aa onto the peptides across the two sample sets does not differ significantly (Figure 4D).

However, despite all the similar patterns regarding the physico-chemical properties of ncPEPs and peptides from coding regions, it is noteworthy that the Chi-square test revealed significant differences regarding the general distribution of aa, their physical-chemical properties, and that of their R-groups (*p* < 0.0001, for all three comparisons). Nevertheless, the comparison of ncPEPs with peptides from known proteins is confounded by the fact that groups of very different sizes were compared. To our knowledge, however, this is the first comparison between human peptides from coding regions and ncPEPs, and our preliminary data suggest several notable differences between these two types of peptides.

### 3.5. Examples of ncPEPs Functions

NcPEPs included in FuncPEP have been shown to play specific roles in many cellular processes, in certain cases even contrary to those played by the ncRNAs that they are translated from. Indeed, the lncRNA, HOXB-AS3, one of the many antisense lncRNAs overlapping the human homeobox B (HOXB) cluster of transcription factor genes first characterized by the FANTOM Consortium [1], plays an oncogenic role in myeloid malignancies and its high expression associates with poor prognosis [35]. On the other hand, its corresponding ncPEP has a tumor suppressive function in colorectal cancer: The loss of this ncPEP is a central tumorigenic event, leading to the reprogramming of cancer metabolism by enabling hnRNP A1-dependent aerobic glycolysis [36].

Additionally, we observed that ncPEPs may act as key regulators in many fundamental processes, including calcium homeostasis [12], gastrulation [37], interaction with the mRNA decapping complex [38], development [39], cell proliferation, muscle growth [40], and immunity [41]. For example, Anderson et al. discovered myoregulin (MLN), responsible for SERCA inhibition. SERCA is a membrane pump controlling the uptake of Ca^2+^ into the sarcoplasmic reticulum (SR). MLN impedes Ca^2+^ uptake by direct interaction with SERCA, finally controlling muscle relaxation. In mice, the genetic deletion of this ncPEP enhances Ca^2+^ retention in skeletal muscles improving exercise performance [12]. Cai et al. demonstrated the role of a not yet named ncPEP, encoded by the lncRNA-Six1. This ncPEP activates the Six1 gene, while the corresponding lncRNA carries out a cis-acting regulation of the protein Six1. Taken together, these data suggest that this ncPEP promotes cell proliferation and is involved in muscle growth [40].

An ncPEP encoded by lncRNA 1500011K16Rik (*Mus musculus*) or LINC00116 (*Homo sapiens*) was published in parallel by two different groups. Mitoregulin or micropeptide regulator of β-oxidation (MOXI) is localized at the inner mitochondrial membrane. By association with the trifunctional protein, it positively influences mitochondrial fatty acid β-oxidation. Mitroregulin knock-out mice suffer from a perturbed fatty acid metabolism and reduced mitochondrial respiratory efficiency, resulting in decreased agility of the animals [42,43]. Interestingly, another human peptide was described by two different groups. The non-annotated *p*-body dissociating polypeptide (NBDY) was first described to be involved in the 5′ to 3′ mRNA decay pathway by interacting with mRNA decapping enzymes [38]. Later the same year, the peptide was also detected to be involved in the immune response upon viral infection [41]. This bifunctionality stresses that ncPEPs are important regulators and not merely translational artefacts.

Given ncPEPs’ roles in fundamental cellular mechanisms, their function in oncogenic development and progression appears probable. Indeed, several studies on ncPEPs have shown that these peptides actually play key roles in a number of human cancers. Many ncPEPs have been found to play protumorigenic or antitumorigenic functions by affecting cancer metabolic reprogramming, the stability of oncogenic proteins, or the epithelial-mesenchymal transition (EMT) process. Zheng et al. showed that the circular RNA circPPP1R12A contains an ORF encoding a functional ncPEP, named circPPP1R12A-73aa. They found that this ncPEP promotes colorectal cancer growth and metastasis via the activation of the Hippo-YAP signaling pathway [44]. Another ncPEP, CASIMO1, promotes breast cancer progression by various mechanisms. It is involved in actin reorganization and thereby facilitates cell mobility. Additionally, it positively influences cell cycle progression and interacts with squalene epoxidase (SQLE), resulting in a modulated steroid synthesis [45]. Other ncPEPs promote tumor progression by stimulating cell proliferation—circE7 [46], influencing immune tolerance in melanoma cells—meloe-3 [47], or inhibiting apoptosis in esophageal squamous carcinoma by interaction with Yin Yang 1—YY1BM [48].

In contrast to that, a number of ncPEPs in our database have tumor-suppressive capacities. CIP2A-BP, for example, encoded by LINC00665, binds directly to the oncogene CIP2A. Upon this interaction, the PIK3A/AKT/NFκB pathway is inhibited, resulting in decreased expression of matrix metallopeptidase (MMP2), MMP9, and Snail that all can promote tumor progression in triple-negative breast cancer (TNBC) [49]. Additionally, in TNBC, a small regulatory peptide of STAT3 (ASRPS), binds to STAT3 and thereby reduces expression of vascular endothelial growth factor (VEGF), resulting in decreased angiogenesis [50]. Furthermore, the micropeptide-inhibiting actin cytoskeleton (MIAC) exerts its tumor-suppressive capacity by inhibiting cell proliferation and metastasis through actin formation suppression in head and neck squamous cell carcinoma [51]. The HOXB-AS3 peptide prevents metabolic reprogramming in colorectal cancer [36], and the ncPEP LINC87aa, derived from a circRNA, inhibits the transcriptional elongation of multiple oncogenes in glioblastoma [52].

**Figure 5:**
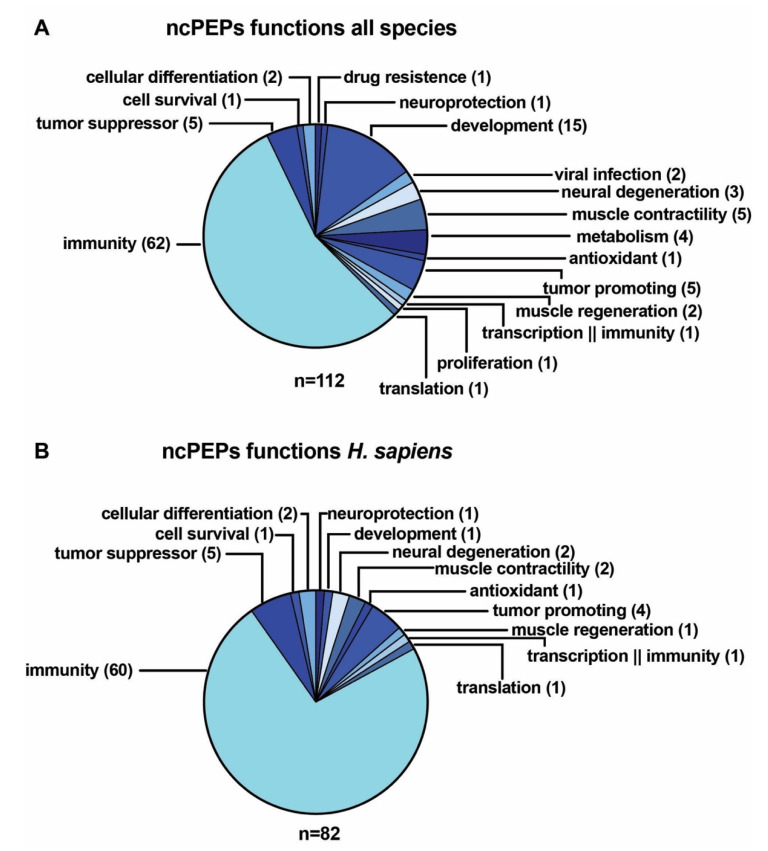
Overview of ncPEP functions. (**A**) Functions of ncPEPs across all species in our database. (**B**) Functions of the human ncPEPs in our database.

An overview of the diverse functions exerted by the ncPEPs in our database reveals that across as all species the majority of ncPEPs is involved in immunity (*n* = 62, 55.36%). This observation is in accordance with the fact that small peptides are capable to bind and regulate the folding and function of histocompatibility leukocyte antigen (HLA) [53,54]. Therefore, we consider that an ingenious method to discover new functional ncPEPs is to pull-down HLA molecules and to analyze the peptides located in their specific binding grooves. Especially in *Drosophila melanogaster* and several plants, ncPEPs are also involved in development, representing the second largest functional group across all species (*n* = 15, 13.39%). Other ncPEPs are involved in different muscular processes (*n* = 7, 6.25%), cancer biology (*n* = 9, 8.04%), neural processes (*n* = 4, 3.57%), metabolism (*n* = 4, 3.57%), and other cellular functions, such as viral infection (*n* = 2, 1.79%), differentiation (*n* = 2, 1.79%), transcription (*n* = 1, 0.89%), translation (*n* = 1, 0.89%), cell survival (*n* = 1, 0.89%), cellular proliferation (*n* = 1, 0.89%), drug resistance (*n* = 1, 0.89%), and one ncPEP has antioxidative capacities (0.89%) (Figure 5A). Since the majority of the ncPEPs in our database are found in *Homo sapiens*, the distribution of functions in the human ncPEP set is similar. Apart from the majority of human ncPEPs being involved in immunogenic processes (*n* = 60, 73.17%), several human ncPEPs play roles in cancers, either working as tumor suppressors (*n* = 5, 6.1%) or exerting oncogenic functions (*n* = 4, 4.89%). A similar number of human ncPEPs are involved in neural (*n* = 3, 3.66%) and muscular processes (*n* = 3, 3.66%), while the others influence development (*n* = 1, 1.22%), transcription (*n* = 1, 1.22%) and translation (*n* = 1, 1.22%), cell survival (*n* = 1, 1.22%), cellular differentiation (*n* = 2, 2.44%), and one has antioxidative effects (Figure 5B). The proportion of immunity-associated ncPEPs is greater in human than in all species, but this difference may be due to ascertainment bias (some families of short immune peptide genes are better-studied in humans) not to a genuine functional distinction. This overview demonstrates that ncPEPs are involved in a multitude of different cellular processes in *Homo sapiens* and other species, implying that they represent an important group of molecules that has not been explored yet. Because of their functions and key roles in cancer initiation and progression, ncPEPs may become a new class of biomarkers and, in cases where they are directly functional, may be amenable to repurposing into therapeutic targets or tools.

## 4. Discussion

The originality of the FuncPEP database is implicit in its three key aspects. First, all included ncPEPs are experimentally confirmed, and not merely predicted. Several studies that we identified during the screening process only employed computational predictions, or indirect experimental methods, to detect potential ncPEPs en-masse, but those candidate ncPEPs were not further validated. These ncPEPs were hence not included in FuncPEP, and will be added in the future only if they become experimentally confirmed. We consider that prediction tools should only be used as an initial step for detecting potential ncPEPs, which needs following experimental confirmation, and we caution against the widespread overreliance on ribosome profiling as an ncPEP discovery pipeline without validations by complementary independent methods.

We observed, during the screening process, multiple ways to study the protein-coding potential of ncRNAs. The phyloP score is an interesting method to detect potential ncPEPs by analyzing the conservation of the genomic sequence [55]. Given that, for known protein-coding genes, a high conservation score is correlated with a higher translation potential [56], many researchers have chosen to use the phyloP score to predict if an ncRNA encodes a peptide, even though unbiased genome-wide phyloP surveys of ncPEPs have not yet been undertaken. Another method frequently used to detect potential ncPEPs is PhyloCSF. This technique allows a specific and easy phylogenic classification of small genomic regions [10]. Based on a formal statistical comparison of phylogenetic codon models, PhyloCSF is a comparative genomics method analyzing multispecies nucleotide sequence alignment to determine whether a genomic region is likely to represent a conserved protein-coding region. However, an important limitation of phyloP and PhyloCSF is that, in lineages as diverse as yeast and mammals, most lncRNA genes, dramatically and unlike protein-coding genes, are not conserved [5]. Even between closely related species, such as the great apes and the prosimians, lncRNA conservation is mostly lacking. In fact, two-thirds to three-quarters of human lncRNAs are not conserved beyond primates [57,58]. Therefore, it is crucial to also employ conservation-independent methods for the discovery of smORFs in ncRNAs. Computational ORF finders [17,18] can identify smORFs of 100 aa or fewer that are able to encode peptides. SmORFs are usually excluded from proteome annotation, especially if they are not conserved and do not represent well-known small proteins, even if they are bound by cellular ribosomes or actively undergo translation.

After ncPEPs are predicted, or as an experimental alternative to computational prediction, two key high-throughput whole-genome laboratory-based methods are currently in wide use to empirically confirm smORFs: ribosome profiling and MS. Ribosome profiling is one of the most popular techniques to assess the translational relevance of smORFs [59]. Based on high-throughput sequencing of the polysome-bound RNA fraction (as opposed to all RNA in the cell), followed by bioinformatics analysis of the mapped RNA fragments, ribosome profiling gives a “global snapshot” of the “translatome” at a particular time point [59]. However, this technique provides only indirect evidence of translation, given that ribosome binding of lncRNAs was observed previously and suggested to also regulate translation of mRNAs by occupying ribosomes. Additionally, one tRNA from a bacterium [60] and a small ncRNA from yeast [61] were shown to exert regulatory capacities in translation in stress conditions, indicating that ncRNAs of all sizes can bind to ribosomes without being translated themselves [62]. Hence, the results from ribosome profiling could represent unproductive encounters between ribosomes and RNAs that do not necessarily result in the translation of the RNAs. More recently, a key advance in bioinformatics analysis of ribosome profiling data has enabled the differential identification of ncRNA smORFs that show clearly mRNA-like patterns of ribosome pausing at the E, P, and A sites of each codon, strongly suggesting that translation is actually occurring [63]. Understanding the proportion of ribosome-lncRNA binding events consistent with E/P/A pausing, as opposed to indiscriminate ribosome sponging by lncRNAs, remains a challenge in the ribosome profiling field. On the other hand, MS is considered the gold-standard technique in proteomics, being the sole direct tool to study the protein-coding potential of ncRNA smORFs. Unlike ribosome profiling, MS has not been commonly used for ncRNA smORF analysis, mainly because of the high cost of the procedure, the necessary extensive customization of proteomics analysis, and the decreased likelihood of detection of low-abundance targets [7,64]. Despite these limitations, MS allows ncPEP detection through the unbiased whole-proteome identification of amino acid sequences in tryptic-digest mass spectra [65], which can then be matched to predicted and known ncRNA smORFs from a custom database.

Second, all ncPEP included in FuncPEP were proven to be functional, being linked to a physiological or pathological phenomenon. Hence, beyond experimental confirmation, the subsequent objective to be achieved when working with ncPEPs is to determine their physiological or pathological function. Targeted genome editing is used to confirm transcriptional relevance, but more importantly to study peptide function by either deleting the entire ORF or deleting or replacing specific parts of the ORF. Subsequently, appropriate phenotypic screens in cell culture or in vivo model organisms are performed. For ncRNAs, disabling (but not deleting) the entire smORF, for example by changing the ATG start codon at the genome level into a codon that does not support translation initiation, is a good way to test whether their cellular function depends on the translation of the smORF. Allowing rapid and efficient genomic editing, CRISPR/Cas9 is one of the most widely adopted “gain and loss of function” techniques [66]. However, also other genome editing techniques are used to analyze ncPEP´s function. For example, Myoregulin, a peptide encoded by a skeletal muscle-specific RNA, previously annotated as an lncRNA, has been studied using Talen (transcription activator-like effector nuclease)-mediated homologous recombination, a powerful and alternative strategy to perform genome editing [12]. Whole-genome high-throughput gene editing functional screens (using CRISPR/Cas9, Zfn, or Talen) in human cell cultures and model organisms is becoming essential for differentiating ncPEP-dependent from RNA-only biological functions of ncRNAs.

Third, we included only ncPEPs that did not exceed 100 aa in length. Our approach is mainly motivated by previous studies that showed that in lncRNAs, most of the ORFs corresponded to peptides less than 100 aa in length [8], as well as by the fundamental definition of an lncRNA as a transcript with solely sub-100-aa ORFs [5]. Moreover, because of their small size, we believe that this class of peptides was overlooked and in the near future will be intensely researched and established as a separate entity.

In particular, putative hormone-like and innate-immunity (defensin-like) functions of ncPEPs, along with the potential of disease-specific ncPEPs to function as signaling molecules and autoantigens, and their possible interactions with pathogens’ nucleic acids and proteins during infection, summarily warrant devoting an increased amount of attention toward the lncRNA proteogenomics field. FuncPEP will facilitate elucidation of this still-emerging biology.

## 5. Conclusions

The FuncPEP database was developed to organize and present the published data on ncPEPs. The first papers on the topic of ncPEPs were published around the year 2000, and we have noticed an exponentially growing interest in ncPEPs, in accordance with the exponentially increasing number of publications and discoveries in the field. Thanks to an easy-to-use interface, FuncPEP allows researchers to find essential, relevant, and up-to-date data on functionally characterized ncPEPs. The database includes details about both the ncPEPs and the related ncRNA transcripts that encode them. Additionally, a link to the original paper reporting the discovery of each ncPEP can be found. Of note, only functionally well-characterized ncPEPs were included in FuncPEP and computationally predicted ncPEPs were excluded and will be included only if their function will be experimentally confirmed. By creating FuncPEP, we aim to provide a useful, easy-to-use, and continually updated web-based bioinformatics tool that allows researchers to quickly and directly access recent discoveries in the area of ncPEPs.

## Availability

FuncPEP is freely accessible at https://bioinformatics.mdanderson.org/Supplements/FuncPEP/.
